# Supplementary material for: Ecological Aspects of Distribution and Population Status Assessment of Rhamnus erythroxyloides subsp. sintenisii (Rich.f) Mabb., a Relict Species in the Kyzylkum Desert of Uzbekistan
Source: Plants (Basel). 2024 Nov 9;13(22):3154. doi: 10.3390/plants13223154 (PMC11597516; doi:10.3390/plants13223154)
Supplement: Supplementary file 1 [file plants-13-03154-s001.zip › plants-3288792-supplementary.pdf]

Supplementary Table S1

Characteristics of plant communities involving *Rhamnus erythroxyloides* subsp. *sintenisii*

Local populations	BUT-1	BUT-2	KUT	AKT
Dominants of plant communities	*Kraschenіnnikovia ceratoides, Rhamnus erythroxyloides* subsp. *sintenisii*	*Krascheninnikovia ceratoides Artemisia diffusa, Rhamnus erythroxyloides* subsp. *sintenisii*	*Rhamnus erythroxyloides* subsp. *sintenisii, Artemisia diffusa, Salsola arbuscula*	*Rhamnus erythroxyloides* subsp. *sintenisii*
Geographic data	N 42.3312<br>E 63.1339<br>h=333 m	N 42.3817<br>E 63.0802<br>h=333 m	N 40.7886<br>E 64.0366<br>h=571 m	N 41.6736<br>E 64.4894<br>h=721 m
Projective coverage, %	12.0	15.0	22.0	15.0
**Species name**	Species abundance (%; +<1%)			
Haloxylon ammodendron (C.A.Mey.) Bunge ex Fenzl	1			
Rhamnus erythroxyloides subsp. *sintenisii* (Rech.f.) Mabb.	3	3	8	5
Salsola arbuscula Pall.			3	
Krascheninnikovia ceratoides (L.) Gueldenst.	4	5		
Artemisia diffusa Krasch. ex Poljakov	2	4	6	2
Ephedra intermedia Schrenk & C.A.Mey.				2
Acanthophyllum elatius Bunge	+			
Ephedra distachya L.		+		
Artemisia juncea Kar. & Kir.			1	2
Astragalus centralis E.Sheld.				+
Scrophularia xanthoglossa Boiss.				+
Anemone petiolulosa Juz.			2	+
Allium borszczowii Regel			+	
Allium kysylkumi Kamelin				+
Carex pachystylis J. Gay.			+	
Gagea bergii Litv.			+	+
Ixiolirion tataricum (Pall.) Schult.& Schult.f.			+	
Ferula karelinii Bunge			+	
Ferula kyzylkumica Korovin				
Lepidium subcordatum		+	+	+

Botsch. et Vved.				
Lagochilus vvedenskyi Kamelin & Tzukerv.				+
Rindera tetraspis Pall.			+	+
Stipa hohenackeriana Trin. & Rupr.	+	1		+
Stipa aktauensis Roshev.				1
Poa bulbosa L.	2	1	2	2
Rheum turkestanicum Janisch			+	
Scariola orientalis (Boiss.) Sojak.			+	+
Tulipa buhseana Boiss.			+	+
Thalictrium isopyroides C.A.Mey.			+	+
Peganum harmala L.	+			
Ranunculus falcatus L.	+		+	+
Eremopyrum bonaepartis (Spreng.) Nevski			+	+
Bromus tectorhum L.	+			+
Diptychocarpus strictus (Fisch. ex M.Bieb.) Trautv.		+		+
Descurainia sophia (L.) Webb ex Prantl	+			
Strigosella turkestanica (Litv.) Botsch.	+	+		
Scandix stellata Banks & Sol.	+	+		
Holosteum umbellatum L.	+	+	+	+
Galium spurium L.			+	
Koelpinia linearis Pall.	+	+	+	+
Arnebia decumbens (Vent.) Coss. & Kralik	+	+		
Lappula microcarpa (Ledeb.) Gürke	+	+		+
Leptaleum filifolium (Willd.) DC.				+
Alyssum desertorum Stapf.	+	+	+	+
Takhtajaniantha pusilla (Pall.) Nazarova		+	+	+
Valerianella szovitsiana Fisch. & C.A.Mey.	+	+	+	+

Veronica campylopoda Boiss.			+	+
Ziziphora tenuior L.	+	+		

Supplementary Table S2

Soil variables in the BSE model for Rh. erythroxyloides subsp. *sintenisii*

Variable code	Variable category	Percentage	Average (*M*) and standard deviation (σ) of the variables in the CAIA area (S_1) and in the econiche (S_2)				*IMd*
			S_1		S_2		
			M	σ^2	*M*	σ^2	
**Characteristics of bedrock soil**							
SSch_90	depth to bedrock (horizon R) up to 200 cm (BDRICM)	2,0	188,74	28,27	196,34	8,21	**1,04**
SSch_91	absolute depth to bedrock (in cm) BDTICM	0,7	4828,22	4818,42	3885,73	2311,16	**0,80**
**Physical soil characteristics**							
SPh_92	bulk density, kg/m^3 (BLDFIE)	0,4	1319,02	246,86	15,79	4,72	**0,01**
SPh_93	mass fraction of sludge (2-50 µm), in % of depth (SLTPPT)	0,3	36,27	8,01	32,24	4,02	**0,89**
SPh_94	rough fragments, volumetric in % of depth (CRFVOL)	1,3	12,90	9,31	15,79	7,72	**1,22**
**Chemical soil characteristics**							
SCh_95	soil ph x 10 in H_2O at depth (PHIHOX)	0,4	74,00	6,68	80,58	1,58	**1,09**
**Soil types in which the development of plant root systems is difficult**							
S_10	Calcic.Solonetz (SN)	0,1	0,66	0,99	1,07	0,93	**1,62**
S_11	Calcic.Vertisols (VR)	0,1	0,53	0,92	0,56	0,74	**1,06**
S_16	Gleyic.Solonetz (SN)	1,6	1,02	1,64	0,01	0,11	**0,01**
S_17	Gypsic.Solonchaks (SC)	7,2	1,23	2,62	0,35	0,59	**0,28**
S_41	Haplic.Leptosols.Eutric (LP)	15,7	1,00	1,84	1,84	1,31	**1,84**
S_42	Haplic.Leptosols (LP)	2,5	2,71	4,81	2,25	1,31	**0,83**
S_50	Haplic.Solonchaks.Sodic (SC)	0,1	0,38	0,9	0,14	0,36	**0,37**
S_51	Haplic.Solonchaks (SC)	0,7	3,49	4,77	2,43	1,91	**0,70**
S_53	Haplic.Vertisols.Eutric (VR)	0,1	0,40	0,81	0,20	0,44	**0,50**
S_58	Lithic.Leptosols (LP)	0,6	0,87	1,78	0,83	1,05	**0,95**
S_63	Mollic.Solonetz (SN)	0,1	0,83	1,32	0,17	0,44	**0,20**
S_65	Rendzic.Leptosols (LP)	2,2	0,65	1,39	1,48	1,33	**2,28**
**Soils characterized by the dynamics of Fe and Al**							
S_02	Aluandic.Andosols (AN)	0,3	0,17	0,61	0,01	0,09	**0,06**

S_06	Calcic.Gleysols (GL)	4,6	1,11	1,52	0,07	0,28	**0,06**
S_14	Endogleyic.Planosols (PL)	0,8	0,45	1,32	0,04	0,22	**0,09**
S_46	Haplic.Planosols.Eutric (PL)	0,2	0,35	0,65	0,003	0,06	**0,01**
S_62	Mollic.Gleysols (GL)	6,6	0,73	1,06	0,03	0,06	**0,04**
**Soils with a well-defined accumulation of organic matter in the upper mineral horizon**							
S_05	Calcic.Chernozems (CH)	0,6	0,66	1,06	0,04	0,21	**0,06**
S_31	Haplic.Chernozems (CH)	0,6	5,15	7,5	0,85	1,11	**0,17**
S_40	Haplic.Kastanozems (KS)	0,7	5,76	8,21	2,92	2,51	**0,51**
S_45	Haplic.Phaeozems (PH)	0,2	3,63	5,68	0,17	0,50	**0,05**
S_61	Luvic.Phaeozems (PH)	0,2	0,60	1,32	0,12	0,34	**0,20**
**Soils with accumulation of moderately soluble salts or other substances**							
S_07	Calcic.Gypsisols (GY)	17,3	1,36	2,22	2,40	1,60	**1,76**
S_23	Haplic.Calcisols.Sodic (CL)	0,5	0,09	0,36	0,02	0,12	**0,22**
S_24	Haplic.Calcisols (CL)	3,6	8,17	8,97	19,54	5,13	**2,39**
S_59	Luvic.Calcisols (CL)	1,2	1,20	1,5	1,71	1,29	**1,43**
S_64	Petric.Calcisols (CL)	0,8	0,44	1,05	0,82	0,93	**1,86**
**Soils with weak or no profile differentiation**							
S_21	Haplic.Arenosols.Calcaric (AR)	0,1	0,41	1,15	0,20	0,43	**0,49**
S_22	Haplic.Arenosols (AR)	2,9	2,59	3,96	1,36	1,55	**0,53**
S_03	Aric.Regosols (RG)	0,6	6,18	6,74	11,45	3,66	**1,85**
S_25	Haplic.Cambisols.Calcaric (CM)	0,1	2,95	2,99	5,40	2,11	**1,83**
S_35	Haplic.Fluvisols.Eutric (FL)	1,2	0,33	0,71	0,11	0,31	**0,33**
S_48	Haplic.Regosols.Dystric (RG)	0,4	0,46	1,12	0,09	0,30	**0,20**
S_56	Leptic.Cambisols (CM)	0,1	0,09	0,42	0,03	0,18	**0,33**
S_57	Leptic.Regosols (RG)	3,0	0,29	0,85	0,03	0,17	**0,10**

Supplementary Table S3

List of 19 bioclimatic variables used in bioclimatic envelope model development. Names and descriptions are based on WorldClim [53]

Variable	Description	Temporal Scale
bio_01	Annual Mean Temperature	Annual
bio_02	Mean Diurnal Range	Variation
bio_03	Isothermality	Variation
bio_04	Temperature Seasonality	Variation
bio_05	Maximum Temperature Warmest Month	Month
bio_06	Minimum Temperature Coldest Month	Month
bio_07	Temperature Annual Range	Annual
bio_08	Mean Temperature Wettest Quarter	Quarter
bio_09	Mean Temperature Driest Quarter	Quarter
bio_10	Temperature Warmest Quarter	Quarter
bio_11	Mean Temperature Coldest Quarter	Quarter
bio_12	Annual Precipitation	Annual
bio_13	Precipitation of Wettest Month	Month
bio_14	Precipitation of Driest Month	Month
bio_15	Precipitation Seasonality	Variation
bio_16	Precipitation of Wettest Quarter	Quarter
bio_17	Precipitation of Driest Quarter	Quarter
bio_18	Precipitation of Warmest Quarter	Quarter
bio_19	Precipitation of Coldest Quarter	Quarter
